# Supplementary material for: Trends in Pseudomonas aeruginosa In Vitro Susceptibility to Ceftolozane/Tazobactam in Latin America: SMART Surveillance Program, 2016–2024
Source: Antibiotics (Basel). 2025 Oct 14;14(10):1018. doi: 10.3390/antibiotics14101018 (PMC12562063; doi:10.3390/antibiotics14101018)
Supplement: Supplementary file 1 [file antibiotics-14-01018-s001.zip › antibiotics-3906663-supplementary.pdf]

SUPPLEMENTARY DATA

Table S1. Number of *P. aeruginosa* isolates collected for the SMART program by Latin American country and clinical site, 2016-2024.

Country/Site[a]	2016	2017	2018	2019	2020	2021	2022	2023	2024	Grand Total
Argentina										
**A**	**47**	**46**	**66**	**41**	**52**	**42**	**39**	**38**	**39**	**410**
B		46	35	41	39	49	42	39	45	336
C			26	60	66	66	53	65	51	387
D	25	46								71
E									14	14
F		16	21	46	35	38	51	54	44	305
Brazil										
A		52	70	42	45	47				256
B	16	17		10	25	15				83
C	40	33	30	31	31	31	27	2		225
D	15									15
E	18	16								34
F	15	12		2	4	3				36
G			23	37	25	33	42	63		223
H						48	41	20		109
I		17	43	61	61	51				233
J		15	25	19	26	36				121
K						48				48
L						17				17
Chile										
**A**	**15**	**34**	**42**	**6**	**14**	**41**	**44**	**53**	**57**	**306**
**B**	**16**	**29**	**36**	**34**	**42**	**39**	**27**	**37**	**28**	**288**
C		63	40	62	46	46	56	59	42	414
Colombia										
**A**	**12**	**21**	**26**	**20**	**21**	**31**	**25**	**35**	**35**	**226**
**B**	**20**	**29**	**53**	**38**	**19**	**25**	**17**	**33**	**28**	**262**
C		4	9	29	23	27	9	10	7	118
D			37	36	32					105
E						62				62
**F**	**19**	**25**	**16**	**19**	**23**	**15**	**22**	**19**	**14**	**172**
**G**	**19**	**26**	**51**	**29**	**54**	**70**	**50**	**52**	**47**	**398**
Dominican Rep.										
A	42									42
B								16	48	64
Ecuador										

A	16				1					17
B		18	22	11	53	46	18	32	29	229
C			14	25	23	36	33	25	26	182
D						36	29	34	31	130
Guatemala										
**A**	**33**	**47**	**29**	**42**	**45**	**28**	**36**	**39**	**47**	**346**
B						12	4	30	35	81
Mexico										
A		25	16	33	31	40	41	43	40	269
**B**	**27**	**30**	**36**	**28**	**31**	**39**	**48**	**23**	**44**	**306**
**C**	**21**	**31**	**20**	**11**	**14**	**27**	**18**	**12**	**22**	**176**
**D**	**60**	**40**	**45**	**41**	**41**	**26**	**43**	**33**	**45**	**374**
**E**	**31**	**28**	**39**	**58**	**39**	**39**	**32**	**49**	**34**	**349**
F				19						19
G		42	25	24	55	42	26	24	36	274
H						54	45			99
I								8	32	40
Panama										
**A**	**47**	**63**	**47**	**50**	**64**	**39**	**65**	**45**	**57**	**477**
**B**	**47**	**46**	**46**	**67**	**46**	**59**	**56**	**58**	**50**	**475**
C			7	6						13
Peru										
A							1	21	23	45
Puerto Rico										
A						14	43	43	39	139
B	48	38	49	39	42	47	35	30		328
C	39									39
D			39	42						81
Venezuela										
A			33	43	43					119
B	7									7
C	45		37	25	48					155
D	39									39
Grand Total	779	955	1,153	1,227	1,259	1,464	1,118	1,144	1,089	10,188

[a] Site information anonymized with letter designation; sites that contributed each year in bold.

Table S2. Longitudinal trends from 2016 to 2024 in the percentage of *P. aeruginosa* isolates identified as multidrug resistant (MDR), difficult-to-treat resistant (DTR), and ceftolozane/tazobactam-susceptible (C/T-susceptible) among (A) all isolates collected in Latin America, and (B) isolates collected from clinical sites that participated in the SMART program each year from 2016 to 2024. Data correspond to Figure 1 in main body of manuscript.

A)

	%		
Year (n)	MDR	DTR	C/T-susceptible
2016 (779)	20.7	15.8	84.9
2017 (955)	20.3	14.6	85.1
2018 (1153)	18.2	15.4	86.5
2019 (1227)	17.3	13.7	85.9
2020 (1259)	18.3	14.3	85.6
2021 (1464)	17.6	13.7	86.4
2022 (1118)	22.1	15.4	85.2
2023 (11445)	16.6	12.2	89.2
2024 (1089)	18.9	13.1	87.1

B)

	%		
Year (n)	MDR	DTR	C/T-susceptible
2016 (414)	27.8	17.9	82.6
2017 (495)	24.1	13.7	86.6
2018 (552)	22.4	13.4	86.2
2019 (484)	16.9	12.0	88.5
2020 (505)	21.1	14.3	87.4
2021 (520)	16.9	12.1	89.1
2022 (522)	23.1	13.0	84.3
2023 (526)	16.9	10.1	88.7
2024 (547)	27.5	13.5	83.9

Table S3. Longitudinal trends from 2016 to 2024 in the percentage of *P. aeruginosa* isolates testing as ceftolozane/tazobactam susceptible stratified by patient age, among (A) all isolates collected in Latin America, and (B) isolates collected from clinical sites that participated in the SMART program each year from 2016 to 2024. Data correspond to Figure 2 in main body of manuscript.

A)

Age group	Year<br>% Susceptible/no. of isolates[a]								
	2016	2017	2018	2019	2020	2021	2022	2023	2024
Pediatric	95.0%/100	92.9%/169	92.9%/155	91.4%/187	90.3%/134	91.1%/158	93.9%/148	92.8%/153	93.3%/150
Adult	83.3%/677	83.5%/781	85.5%/993	85.0%/1017	84.8%/1086	85.7%/ 1263	84.1%/923	88.6%/940	85.7%/896

[a]Age not provided for 159 isolates.

B)

Age group	Year<br>% Susceptible/no. of isolates[a]								
	2016	2017	2018	2019	2020	2021	2022	2023	2024
Pediatric	95.7%/70	99.0%/99	95.7%/92	98.9%/95	94.7%/75	94.7%/95	97.3%/74	98.9%/95	97.1%/103
Adult	79.0%/343	83.5%/393	85.1%/457	86.0%/385	86.2%/426	87.5%/423	82.2%/444	87.0%/431	80.8%/443

[a]Age not provided for 22 isolates.

Table S4. Longitudinal trends from 2016 to 2024 in the percentage of *P. aeruginosa* isolates testing as ceftolozane/tazobactam susceptible stratified by infection source, among (A) all isolates collected in Latin America, and (B) isolates collected from clinical sites that participated in the SMART program each year from 2016 to 2024. Data correspond to Figure 3 in main body of manuscript.

A)

Specimen source[b]	Year % Susceptible/no. of isolates[a]									*p*-value (two tailed)[d]
	2016	2017	2018	2019	2020	2021	2022	2023	2024	
BSI	NA[c]	NA	91%/178	81.9%/149	78.4%/194	86.1%/209	84.8%/171	92.2%/180	89%/181	0.128
IAI	86.6%/186	85.6%/160	84.5%/168	83%/153	89.9%/169	87%/161	89.9%/138	85.7%/133	87.7%/171	0.356
RTI	85.9%/511	85.8%/618	88.6%/659	88.7%/767	87.9%/755	87.6%/911	84.3%/675	89.1%/709	86%/614	0.972
UTI	73.8%/80	82.6%/172	73.1%/145	78.4%/153	78.8%/132	80%/175	85%/133	90.6%/117	88.1%/118	<0.0001

[a]Infection source was not provided for 43 isolates.
[b]BSI, bloodstream infection; IAI, intra-abdominal infection; RTI, respiratory tract infection; UTI, urinary tract infection.
[c]NA=not applicable (bloodstream infection isolates were not collected in 2016 and 2017).
[d]Cochran-Armitage test for trend (XLSTAT v2024.2.2)

B)

Specimen source[b]	Year % Susceptible/no. of isolates[a]									*p*-value (two tailed)[d]
	2016	2017	2018	2019	2020	2021	2022	2023	2024	
BSI	NA[c]	NA	93.3%/89	81.1%/53	84.5%/84	91.8%/98	84.8%/92	89.5%/86	87.1%/93	0.673
IAI	83.3%/120	81.8%/88	84.8%/79	81%/58	91.3%/69	89.2%/65	87.1%/70	90.7%/54	86%/86	0.118
RTI	83%/253	89.6%/318	88.1%/310	93.2%/311	88.1%/295	90.3%/289	83.1%/290	89.2%/315	81.6%/304	0.116
UTI	70%/40	80.7%/88	76.1%/71	78%/59	83.3%/54	77.3%/66	85.7%/70	88.2%/68	86.9%/61	0.011

[a]Infection source was not provided for 19 isolates.
[b]BSI, bloodstream infection; IAI, intra-abdominal infection; RTI, respiratory tract infection; UTI, urinary tract infection.
[c]NA=not applicable (bloodstream infection isolates were not collected in 2016 and 2017).
[d]Cochran-Armitage test for trend (XLSTAT v2024.2.2)

Table S5. Longitudinal trends from 2016 to 2024 in the percentage of *P. aeruginosa* isolates that tested as ceftolozane/tazobactam susceptible, by country among (A) all isolates collected in the Latin American region from countries that participated in the SMART program each year and (B) isolates collected from clinical sites that participated each year from 2016 to 2024. Data correspond to Figure 4 in main body of manuscript.

A)

Country	Year									*p*-value (two tailed)[a]
	2016	2017	2018	2019	2020	2021	2022	2023	2024	
Argentina	84.7%	87.7%	93.2%	93.6%	93.8%	82.1%	94.6%	95.9%	95.3%	0.004
Chile	90.3%	66.7%	71.2%	59.8%	69.6%	77%	73.2%	81.2%	79.5%	0.006
Colombia	87.1%	87.6%	84.9%	78.9%	83.1%	82.6%	84.6%	89.9%	90.1%	0.194
Guatemala	100%	100%	82.8%	100%	86.7%	87.5%	72.5%	73.9%	52.4%	<0.0001
Mexico	72.7%	82.7%	85.6%	84.1%	80.1%	83.1%	76.7%	87.5%	86.6%	0.047
Panama	85.1%	89.9%	86%	97.6%	88.2%	99%	96.7%	98.1%	96.3%	<0.0001
Puerto Rico	86.2%	92.1%	98.9%	87.7%	97.6%	93.4%	92.3%	83.6%	94.9%	0.908

[a]Cochran-Armitage test for trend (XLSTAT v2024.2.2)

B)

Country	Year									*p*-value (two tailed)[a]
	2016	2017	2018	2019	2020	2021	2022	2023	2024	
Argentina	76.6%	84.8%	92.4%	90.2%	88.5%	85.7%	84.6%	94.7%	92.3%	0.076
Chile	90.3%	90.5%	84.6%	92.5%	92.9%	90%	84.5%	91.1%	78.8%	0.145
Colombia	87.1%	87.1%	89.7%	79.2%	85.5%	82.3%	83.3%	89.2%	89.5%	0.738
Guatemala	100%	100%	82.8%	100%	86.7%	92.9%	72.2%	53.8%	25.5%	<0.0001
Mexico	72.7%	76.7%	85%	83.3%	86.4%	87.8%	77.3%	89.7%	89.7%	0.000
Panama	85.1%	89.9%	84.9%	97.4%	88.2%	99%	96.7%	98.1%	96.3%	<0.0001

[a]Cochran-Armitage test for trend (XLSTAT v2024.2.2)
